# Achieving global equity for COVID-19 vaccines: Stronger international partnerships and greater advocacy and solidarity are needed

**DOI:** 10.1371/journal.pmed.1003772

**Published:** 2021-09-13

**Authors:** J Peter Figueroa, Peter J. Hotez, Carolina Batista, Yanis Ben Amor, Onder Ergonul, Sarah Gilbert, Mayda Gursel, Mazen Hassanain, Gagandeep Kang, David C. Kaslow, Jerome H. Kim, Bhavna Lall, Heidi Larson, Denise Naniche, Timothy Sheahan, Shmuel Shoham, Annelies Wilder-Smith, Samba O. Sow, Nathalie Strub-Wourgaft, Prashant Yadav, Maria Elena Bottazzi

**Affiliations:** 1 University of the West Indies, Mona, Kingston, Jamaica; 2 Texas Children’s Center for Vaccine Development, Baylor College of Medicine, Houston, Texas, United States of America; 3 Médecins Sans Frontières, Rio de Janeiro, Brazil; 4 Center for Sustainable Development, Columbia University, New York, New York, United States of America; 5 Koc University Research Center for Infectious Diseases, Istanbul, Turkey; 6 Jenner Institute, Nuffield Department of Medicine, Oxford University, Oxford, United Kingdom; 7 Middle East Technical University, Ankara, Turkey; 8 College of Medicine, King Saud University, Riyadh, Saudi Arabia; 9 Christian Medical College, Vellore, India; 10 PATH, Seattle, Washington, United States of America; 11 International Vaccine Institute, Seoul, South Korea; 12 University of Houston College of Medicine, Houston, Texas, United States of America; 13 London School of Hygiene & Tropical Medicine, London, United Kingdom; 14 ISGlobal-Barcelona Institute for Global Health-Hospital Clinic-University of Barcelona, Spain; 15 University of North Carolina, Gillings School of Global Public Health, Chapel Hill, North Carolina, United States of America; 16 Johns Hopkins University School of Medicine, Baltimore, Maryland, United States of America; 17 Institute of Social and Preventive Medicine, University of Bern, Switzerland; 18 Heidelberg Institute of Global Health, University of Heidelberg, Heidelberg, Germany; 19 Center for Vaccine Development, Bamako, Mali; 20 University of Maryland, Maryland, United States of America; 21 Drugs for Neglected Diseases Initiative, Geneva, Switzerland; 22 Center for Global Development, Washington, DC, United States of America; 23 Harvard Medical School, Boston, Massachusetts, United States of America; 24 Affiliate Professor, Technology and Operations Management, INSEAD, Fontainebleau, France

## Abstract

Peter Figueroa and co-authors advocate for equity in the worldwide provision of COVID-19 vaccines.

Many may not be aware of the full extent of global inequity in the rollout of Coronavirus Disease 2019 (COVID-19) vaccines in response to the Severe Acute Respiratory Syndrome Coronavirus 2 (SARS-CoV-2) pandemic. As of June 20, 2021, only 0.9% of those living in low-income countries and less than 10% of those in low- and middle-income countries (LMICs) had received at least 1 dose of a COVID-19 vaccine compared with 43% of the population living in high-income countries (HICs) [1] (Fig 1). Only 2.4% of the population of Africa had been vaccinated compared with 41% of North America and 38% of Europe [1,2] (S1 Fig). Primarily due to the inability to access COVID-19 vaccines, less than 10% of the population in as many as 85 LMICs had been vaccinated compared with over 60% of the population in 26 HICs [1]. Only 10 countries account for more than 75% of all COVID-19 vaccines administered [3]. This striking and ongoing inequity has occurred despite the explicit ethical principles affirming equity of access to COVID-19 vaccines articulated in WHO SAGE values framework [4,5] prepared in mid-2020, well prior to the availability of COVID-19 vaccines.

**Fig 1:**
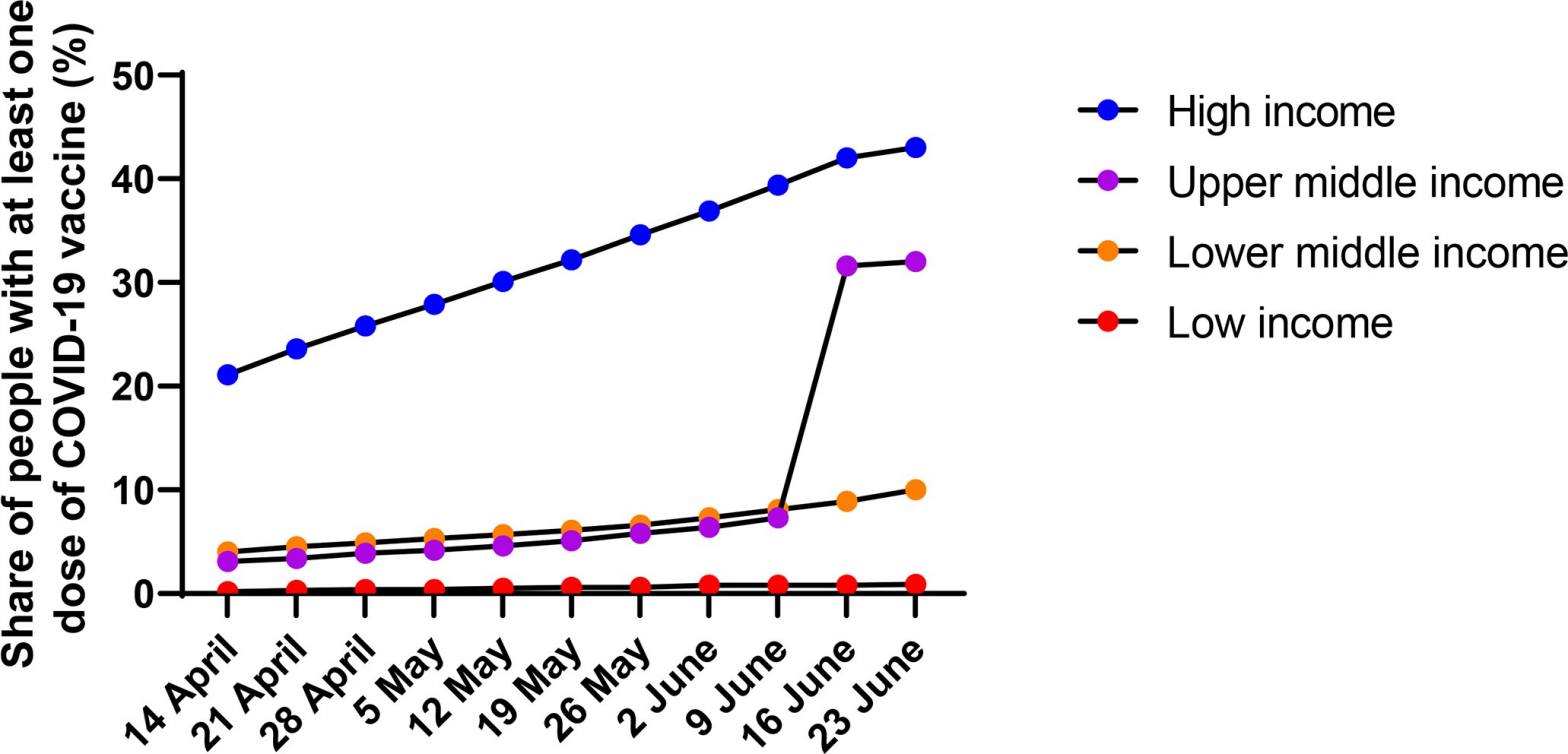
Proportion of people vaccinated with at least 1 dose of COVID-19 vaccine by income (April 14 to June 23, 2021). Note: Data on China appeared on the database on June 9, hence the jump in upper middle-income countries. COVID-19, Coronavirus Disease 2019. *Source*: *https://ourworldindata.org/covid-vaccinations*.

The COVID-19 pandemic highlights the grave inequity and inadequacy of the global preparedness and response to serious emerging infections. The establishment of the Coalition for Epidemic Preparedness Innovations (CEPI) in 2018, the Access to COVID-19 Tools Accelerator (ACT-A), and the COVID-19 Vaccines Global Access (COVAX) Facility in April 2020 and the rapid development of COVID-19 vaccines were all positive and extraordinary developments [6]. The COVAX Facility, as of June 2021, has delivered approximately 83 million vaccine doses to 75 countries, representing approximately 4% of the global supply, and one-fifth of this was for HICs [7]. The COVAX Facility has been challenged to meet its supply commitments to LMICs due to insufficient access to doses of COVID-19 vaccines with the prerequisite WHO emergency use listing (EUL) or, under exceptional circumstances, product approval by a stringent regulatory authority (SRA) [8,9]. Because of the anticipated insufficient COVID-19 vaccine supply through the COVAX Facility, the majority of nonvaccine-producing LMIC countries made the decision, early in the COVID-19 pandemic, to secure and use vaccines produced in China or Russia prior to receipt of WHO EUL or SRA approval. Most of the vaccines used in LMICs as of June 20, 2021 (nearly 1.5 billion doses of the 2.6 billion doses administered) were neither WHO EUL or SRA approved at the time they were given [10]. This may raise possible concerns with respect to the effectiveness, safety, and acceptability of individual vaccines used by many countries [8,9].

## G7 leaders fall short

Although the recent declaration of G7 leaders to donate 1 billion vaccine doses [11] over the next year was welcome news, the donation falls far short of the more than 11 billion doses WHO estimates are required to accelerate control of the pandemic and avert millions of preventable deaths globally due to COVID-19. The G7 leaders failed to lead or even initiate a meaningful roadmap, nor pledge the necessary resources to support the implementation, to accelerate global access and equity to COVID-19 vaccines, in addition to other measures to reduce mortality and control the pandemic. While HICs contributed to the formation and funding of COVAX and the COVAX Facility responsible for equitable global access of COVID-19 vaccines, bilateral contracts with the pharmaceutical companies have monopolized most of the available vaccines [2,12]. A stark example is the case of the Indian vaccine manufacturers, which had to redirect their previously committed vaccine supplies to address the massive surge of COVID-19 cases in India during the second quarter of 2021 [2].

## An international initiative to support vaccine technology transfer is needed

The governments of South Africa and India have called for the waiver of intellectual property protections for patents, industrial designs, trade secrets, and regulatory data for COVID-19 vaccines and therapies. The United States President Biden supported the Trade-Related Aspects of Intellectual Property Rights (TRIPS) waiver call as have China and Russia [12,13]. Of urgent critical importance, however, is technology transfer to enable more vaccine manufacturers to produce vaccines under license from the vaccine originators, largely pharmaceutical companies. Along these lines, the World Trade Organization (WTO) has proposed the use of voluntary licensing arrangements, led by public–private partnerships, that would enable the transfer of high-quality know-how needed to produce safe, high-quality, and effective vaccines [14]. For this to be successful, there must be a fully funded, internationally coordinated initiative that facilitates technology transfer, building of vaccine manufacturing, scientific and regulatory capacity in different regions, and a genuine commitment to working collectively in the common interest that transcends national boundaries and narrow interests [13,15,16].

## Improve vaccine access to low- and middle-income countries

While international capacity building and strengthening is essential, we also must redouble the efforts to leave no one behind by providing COVID-19 vaccine access to all the world’s LMIC populations now. Many HICs can make more vaccine doses available sooner than promised without compromising their ability to vaccinate their own populations. This requires a 2-pronged initiative. First, maximize vaccine donations from HICs and the pharmaceutical companies through COVAX. Countries such as the US could step up their efforts by leveraging US funding and resources to enhance the impact of COVAX and support a roadmap for immediate distribution of currently unallocated or reserve doses of vaccines [17,18]. Second, embark on a parallel initiative to ramp up production and distribution capacity for additional doses of vaccines. Based on the estimated 3 billion people who live in LMICs, this means the scale-up and manufacture of 6 billion doses, preferably during 2021 [19]. For this to happen, we need a full inventory of all mRNA and adenovirus-vectored vaccines currently available, understand the commitments to produce more of these vaccines in the coming months, and then fill that substantial gap with new recombinant protein-based vaccines now being produced in India, China, US/Europe, and elsewhere [10,20–22]. Such recombinant protein-based vaccines can be easily scaled up and delivered, with prospects of high efficacy against the variants of concern, as seen with at least 1 protein-based vaccine [23]. This step is essential to halt the spread of variants globally and the high death tolls anticipated in Africa, Latin America, and Southeast Asia.

A few high-income or well-positioned middle-income countries have made significant progress in vaccinating their populations; however, the global response to the COVID-19 pandemic continues to fall gravely short of what is possible and required to reduce mortality and morbidity. Until urgent measures are taken, the most vulnerable living in LMICs will remain excluded from global health progress, exacerbating inequities (Box 1). It is important to recognize that the bulk of vaccines now in use would not have been developed without significant governmental and multilateral investments. Moving forward, we need sustainability, with substantial pandemic preparedness funding for international agencies to support global public health and research. Governments should preserve a share in the patents of pharmaceutical companies when government support has made a tangible contribution to the development of the product being patented. At the same time, people in LMIC need to hold their leaders more accountable to ensure that they advocate and negotiate on their behalf more effectively and form alliances that can make meaningful gains. Key stakeholders must continue to learn the lessons, forge new initiatives and partnerships, and advocate for tangible actions that promote greater equity, justice, and solidarity.

**Box 1. Key priorities and initiatives for achieving global equity for COVID-19 vaccines**

- Immediate distribution of unallocated or reserve doses of vaccines and donations through the COVAX Facility
- Ramp up production and distribution capacity for additional doses of vaccines
- Technology transfer to enable more vaccine manufacturers to produce vaccines under license from the vaccine originators
- A fully funded, internationally coordinated initiative that facilitates technology transfer, building of vaccine manufacturing, and scientific and regulatory capacity in different regions
- Governments should preserve a share in the patents of pharmaceutical companies when their support has made a tangible contribution to the development of the product being patented
- Some of the funds arising from these shares could support key multilateral agencies and invest in better pandemic preparedness at global and national levels
- These initiatives require genuine commitment to working collectively in the common interest to promote global equity to COVID-19 vaccines and pandemic preparedness

## Supporting information

S1 Fig Proportion of people vaccinated with at least 1 dose of COVID-19 vaccine by continent (April 14 to June 23, 2021). Note: Data on China appeared on the database on June 9, hence the jump in upper middle-income countries. COVID-19, Coronavirus Disease 2019. *Source*: *https*:*//ourworldindata*.*org/covid-vaccinations*.
(TIF)

## Abbreviations

ACT-A: Access to COVID-19 Tools Accelerator
CEPI: Coalition for Epidemic Preparedness Innovations
COVAX: COVID-19 Vaccines Global Access
COVID-19: Coronavirus Disease 2019
EUL: emergency use listing
HIC: high-income country
LMIC: low- and middle-income country
SARS-CoV-2: Severe Acute Respiratory Syndrome Coronavirus 2
SRA: stringent regulatory authority
TRIPS: Trade-Related Aspects of Intellectual Property Rights
WTO: World Trade Organization
