# Supplementary figures and images for: Achieving global equity for COVID-19 vaccines: Stronger international partnerships and greater advocacy and solidarity are needed
Source: PLoS Med. 2021 Sep 13;18(9):e1003772. doi: 10.1371/journal.pmed.1003772 (PMC8475996; doi:10.1371/journal.pmed.1003772)

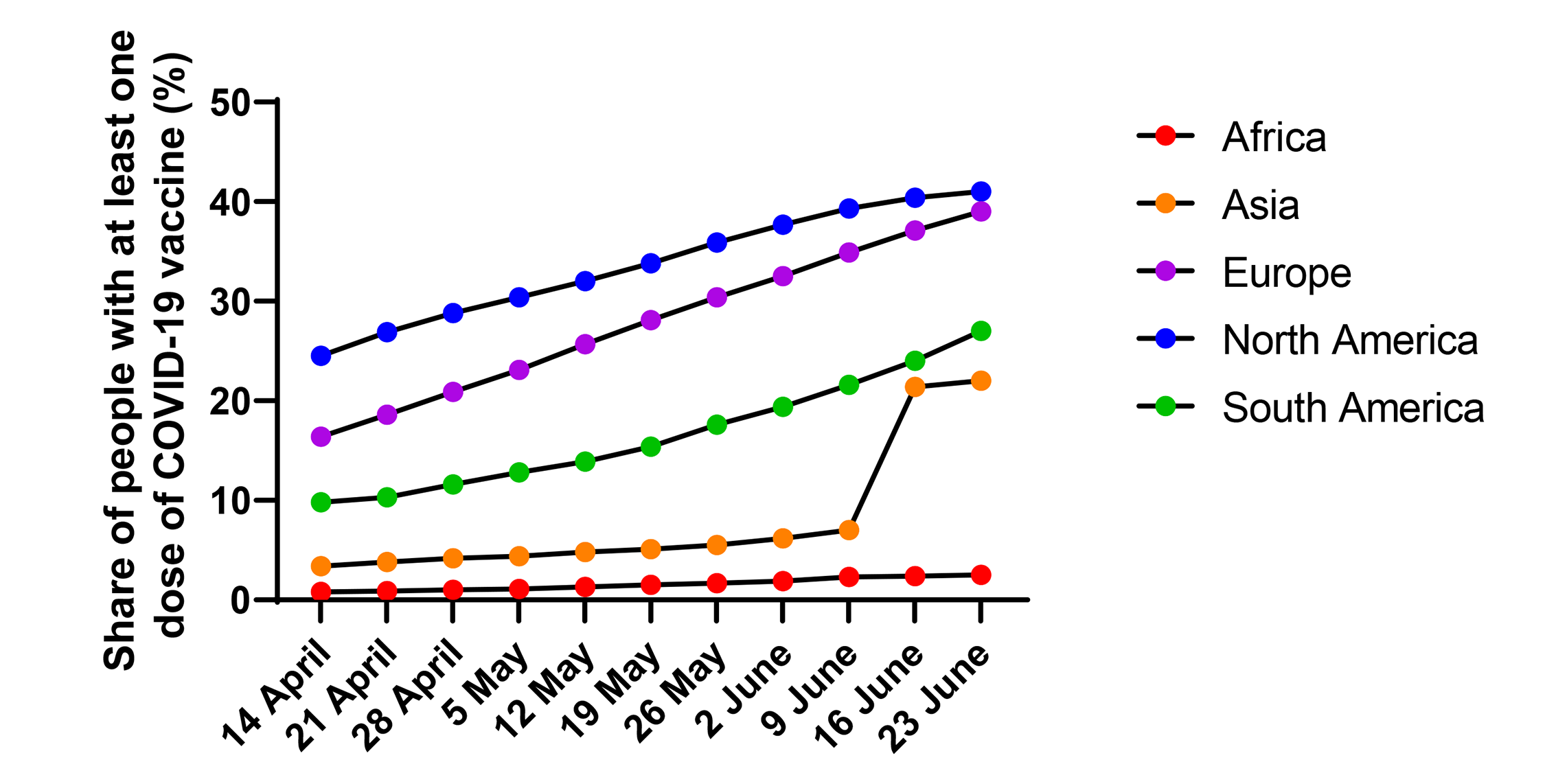

Supplement: S1 Fig — Note: Data on China appeared on the database on June 9, hence the jump in upper middle-income countries. COVID-19, Coronavirus Disease 2019. Source: https://ourworldindata.org/covid-vaccinations. (TIF) [file pmed.1003772.s001.tif]
